# Isolation and Characterization of a Conserved Domain in the Eremophyte H^+^-PPase Family

**DOI:** 10.1371/journal.pone.0070099

**Published:** 2013-07-29

**Authors:** Yanqin Wang, Shuangxia Jin, Maojun Wang, Longfu Zhu, Xianlong Zhang

**Affiliations:** 1 National Key Laboratory of Crop Genetic Improvement, Huazhong Agricultural University, Wuhan, Hubei, China; 2 College of Life Science, Tarim University, Alaer, Xinjiang, China; East Carolina University, United States of America

## Abstract

H^+^-translocating inorganic pyrophosphatases (H^+^-PPase) were recognized as the original energy donors in the development of plants. A large number of researchers have shown that H^+^-PPase could be an early-originated protein that participated in many important biochemical and physiological processes. In this study we cloned 14 novel sequences from 7 eremophytes: *Sophora alopecuroid* (*Sa*), *Glycyrrhiza uralensis* (*Gu*), *Glycyrrhiza inflata* (*Gi*), *Suaeda salsa* (*Ss*), S*uaeda rigida* (*Sr*), *Halostachys caspica* (*Hc*), and *Karelinia caspia* (*Kc*). These novel sequences included 6 ORFs and 8 fragments, and they were identified as H^+^-PPases based on the typical conserved domains. Besides the identified domains, sequence alignment showed that there still were two novel conserved motifs. A phylogenetic tree was constructed, including the 14 novel H^+^-PPase amino acid sequences and the other 34 identified H^+^-PPase protein sequences representing plants, algae, protozoans and bacteria. It was shown that these 48 H^+^-PPases were classified into two groups: type I and type II H^+^-PPase. The novel 14 eremophyte H^+^-PPases were classified into the type I H^+^-PPase. The 3D structures of these H^+^-PPase proteins were predicted, which suggested that all type I H^+^-PPases from higher plants and algae were homodimers, while other type I H^+^-PPases from bacteria and protozoans and all type II H^+^-PPases were monomers. The 3D structures of these novel H^+^-PPases were homodimers except for *SaVP3*, which was a monomer. This regular structure could provide important evidence for the evolutionary origin and study of the relationship between the structure and function among members of the H^+^-PPase family.

## Introduction

A basic property of life is the ability of an organism to regulate cellular pH and ion homeostasis for its normal growth and development. The concerted action of H^+^-translocating enzymes (H^+^-pumps) and cation/H^+^ exchangers are vital to establish and maintain optimal ion and pH gradients. These gradients exist between the cytoplasm and vacuole and between the cytoplasm and rhizosphere and are essential for cell function and plant development [1], [2], [3]. Since the 1940s, it has been proposed that the family of vacuolar membrane H^+^-PPases generate proton gradients in endomembrane compartments by using pyrophosphate (PPi) instead of ATP to act as a biological energy donor [4], [5], [6]. As the energy-rich phosphate product of early bacterial photophosphorylation, PPi was assumed to arise earlier than ATP in the origin and evolution of life as determined by changing the growth conditions of *R. rubrum* from aerobic/dark to anaerobic/light [7], [8], [9], [10], [11]. The H^+^-proton-pumping inorganic pyrophosphatase (H^+^-PPase) family uses PPi rather than ATP for energy coupling and utilization in biological membranes. It was proved that H^+^-PPase couples formation of PPi from Pi in light, hydrolyzes PPi to Pi and transfers energy in dark in the photosynthetic bacterium *Rhodospirillum rubrum* [12], [13]. It was inferred that H^+^-PPase could be the early origin of the acidocalcisomes [14], which are characterized by their acidic nature, high electron density, high concentration of calcium, magnesium, and other elements in addition to pyrophosphate (PPi) and poly P [7]. The acidocalcisome may have appeared earlier than the divergence of the superkingdoms of life (Archaea, Bacteria and Eukarya) based on the analysis of function and the evolutionary dynamics of its domains [9], [15]. H^+^-PPases use PPi-energy to pump H^+^ into the acidocalcisome and produce electrochemical gradients, so H^+^-PPase was regarded as the specific protein that promotes accumulation of Ca^2+^ and other ions in acidocalcisome [16]. Seufferheld *et* *al*. [9] investigated the divergence of protein domains in the H^+^-PPase molecules, and domain PF03030 was found to be shared by 31 species in Eukarya, 231 in Bacteria, and 17 in Archaea. This domain is associated with the function of H^+^-PPase, namely, to hydrolyze diphosphate to phosphate and H_2_O or synthesize diphosphate using phosphate as a substrate. This suggests that the domain and the enzyme were already present in the Last Universal Common Ancestor (LUCA). So it was inferred that H^+^-PPase could be an early origin protein related to the acidocalcisome because it was demonstrated that the H^+^-PPase is ubiquitous in some algae, protozoans, bacteria and archaebacteria [17].

Native H^+^-PPases are divided into two types (type I and type II) according to whether they are K^+^-dependent or K^+^-independent. So far the most K^+^-independent H^+^-PPases were identified in eukaryotes, bacteria and archaea, whereas K^+^-dependent H^+^-PPases were identified only in eukaryotes [18]. However, this observtion was contradicted when H^+^-PPase was found in the heterotrophic euglenoid *A. longa* [19], *A. thaliana* [20] and the Apicomplexan *P. falciparum* [21], both of which clearly clustered with the bacterial K^+^-independent H^+^-PPases. In the plant and algae, K^+^-dependent and K^+^-independent H^+^-PPases showed different subcellular location; the former is located on tonoplast membrane [22], [23], and the latter is located on Golgi-endoplasmic reticulum membranes [6], [24]. All the available evidence is consistent with the notion that suggests that most protist H^+^-PPase constitute a compact cluster and that these organisms are rather close in evolutionary terms. However, it is possible that different evolutionary histories or lateral gene transfers between H^+^-PPases of different (micro) organisms cannot be ruled out, and therefore, led to K^+^-independent H^+^-PPases in higher plants [20], [25]. This conclusion contributes to the clarification of the evolutionary relationship between members of the gene family, even between different organisms. Three conserved domains existed in all type I and type II H^+^-PPase, they are GGGIFTKCADVGADLVGKVEAGIPEDDPRNPAVIADNVGDNVGDCAGMAADLFETY [26], GNTTAA [6], and EYYT [25].

Although a mass of members of the H^+^-PPase family were characterized and their conserved domains were inferred, there are two notable points worth mentioning. One is that obtaining high-resolution 3D information of the H^+^-PPase has been unsuccessful, and the other is the unknown environmental effect on the gene structure of H^+^-PPase. With the development of bioinformatics, most 3D structures of proteins have been predicted. Accurate 3D structure prediction is conducive to inferring correct function and evolution. Evolution is directly affected by environment. Eremophytes develop under conditions of high saline-alkali soil, extreme drought, extreme temperature and strong long duration illumination, and they have evolved a tolerance to abiotic stress. Comparing the diversity of genes between eremophytes and glycophytes will be beneficial for studying the eremophyte tolerance to abiotic stresses.

In our research, we isolated and characterized the H^+^-PPases of eremophytes from Xinjiang, a desert region in northwestern China. Six ORFs and 8 fragments of H^+^-PPase from the eremophytes *Sophora alopecuroid* L. (*Sa*), *Glycyrrhiza uralensis Fisch* L. (*Gu*), *Glycyrrhiza inflata Batalin* L. (*Gi*), *Suaeda salsa* L. (*Ss*), S*uaeda rigida* Kung et G. L. (*Sr*), *Halostachys caspica* L. (*Hc*) and *Karelinia caspia* (Pall.) L. (*Kc*) were cloned. At the same time, 17 identified type I *H^+^-PPase* genes from typical higher plants; 8 identified type I *H^+^-PPase* genes from algae, bacteria and protozoans; and 9 identified type II *H^+^-PPase* genes were analyzed with 14 novel clones of *H^+^-PPase* genes. Their homology, conserved motifs, function, and 3D structures were analyzed and a phylogenetic tree was constructed. This finding could provide reference for illustrating the evolution among *H^+^-PPase* members and also for evaluating the potential use in improving stress resistance of crops.

## Materials and Methods

### 1. Materials Used for Cloning *H^+^-PPase* Homologues and their Natural Habitat

The seeds of following 7 eremophytes were collected from the Alaer environment just around Tarim University, where the first author works, in the Tarim Basin. It is an open and public area, not included in a protected park or private land. And the plants we collected are grown in a wild condition and not included in endangered or protected species in this environment. So no specific permissions were required for these locations/activities. In our study, no animal experiment was involved.

H^+^-PPase homologs were obtained from 7 species of eremophytes from the Tarim Basin around Tarim University, Xinjiang, China. These eremophytes were *Sophora alopecuroides* L. (*Sa*), *Glycyrrhiza uralensis Fisch* L. (*Gu*), *Glycyrrhiza inflata Batalin* L. (*Gi*), *Suaeda salsa* L. (*Ss*), S*uaeda rigida* Kung et G. L. (*Sr*), *Halostachys caspica* L. (*Hc*), and *Karelinia caspia* (Pall.) L. (*Kc*). The first three species belong to Leguminosae, the next three are from Chenopodiaceae, and the last one belongs to Compositae. The seeds of 7 eremophytes were collected from the Alaer environment in the Tarim Basin and were germinated for three weeks. And then the leaves and roots were gathered, respectively, and were put into liquid nitrogen and stored at −80°C till for RNA isolation. Alaer is located at longitudes 80° 30′ to 81° 58′ east, latitude 40° 22′ to 40° 57′ north, and belongs to the warm temperate desert and arid climate. The annual sunshine duration is approximately 1,556∼ 2,992 hrs, with annual average temperature between 8.9∼ 11.4°C. The highest temperature is above 45°C with annual precipitation of only 42∼ 76 mm, and the average annual evaporation (potential) reaches 1,900∼ 2,800 mm. The relative humidity is less than 5% daytime during the summer, and the soil salt is between 1∼ 10%, pH 7.5∼ 9.5. The 7 species and their habitat are shown in Figure S1. Of these plants, *Suaeda salsa* is a therophyte while the others are perennial plants.

### 2. RNA Isolation and *H^+^-PPase* Genes Cloning

Seeds of the eremophytes were germinated at room temperature under a light period of 16 h/8 h day/night for 3 weeks. Total RNA was extracted from leaves and roots of the 7 eremophytes, using the method described by Zhu *et al*. [27]. Total RNA (2 µg) was reverse-transcribed into cDNA using the SuperScript III reverse transcriptase (Invitrogen, Carlsbad, CA, USA), the cDNA products were diluted to 200 µL which were used as templates in the following PCR. To clone the *H^+^-PPase* genes from the experimental plants, degenerate primers for PCR were designed according to the sequence of the conserved domain region of known *H^+^-PPase* genes from NCBI (http://blast.ncbi.nlm.nih. gov/). The RT-PCR mixture contained: 2.5 µL 10× PCR buffer, 0.3 µL Easy-Taq DNA polymerase (5 u/µL, Transgen, Beijing, China), 0.3 µL dNTP (10 mM), 0.3 µL appropriate paired primers (10 µM), 1 µL cDNA products, and ddH_2_O added to 25 µL. Thermal cycle parameter was: 94°C for 5 min, followed by 35 cycles of 94°C for 40 s, 58°C for 40 s, and 72°C for 90 s. The PCR products were cloned into the pEasy-T3 Cloning vector (Transgen, Beijing, China) and sequenced. The rapid-amplification of cDNA ends (RACE) templates and adapter primers were prepared following the GeneRacer™ Kit user manual (Invitrogen). All primers are listed in Table 1.

**Table 1:** Primers for RACE and ORF of the H^+^-PPases from the 7 eremophytes.

species	Primer name	Primer sequence	Destination
For all species	degenerate primer-up	GGHGGYATYTACACBAARGC	H^+^-PPase gene cloning
	degenerate primer-down	GGCDGCVGARCCRATGGC	H^+^-PPase gene cloning
For all species	5R1	TTCACCCCAAAAGAAGAGATGGAAGC	5′- RACE
	5R2	CATTGTCAGCAATCACAGCTGGATTTC	5′- RACE
	3R1	TCTTTGGTGCCTTTGTGAGCCGAGC	3′- RACE
	3R2	GGTGGTGCTTGGGATAATGCCAAG	3′- RACE
*Sa*	SaVP1-1	ATGGGTGCATCCATTCTCCCAGATCTCG	SaVP1 gene cloning
	SaVP1-2	TTAGATCTTGAAGAGTAAGCCACCATGT	
*Gu* and *Gi*	GuVP1	ATGGGAGCAGCGATTCTCCCAGATCTCG	GuVP/GiVP gene cloning
	GuVP2	TTAGATTTTGAAGAGTAGGCCACCGT	
*Kc*	KcVP1	ATGGGTGCATCCATTCTCCCAGATCTCG	KcVP1 gene cloning
	KcVP2	TTAGATTTTGAAGAGTAGGCCACCGT	

### 3. Homology, Phylogeny and 3D Structural Analysis of the *H^+^-PPase* Genes

The identified H^+^-PPase protein sequences were collected from the NCBI (http://blast.ncbi.nlm.nih. Gov/), and homology analysis of the sequences was compared using BLASTx of NCBI online. Transmembrane regions of *SaVP1* were predicted using TMHMM online (http://www.expasy.ch/tools/). A phylogenetic tree was constructed using the Neighbor-Joining method by CLUSTAL X1.83 and MEGA 4.1 software. Conserved domains and motifs were predicted by CLUSTAL X1.83 and Logo soft online (http://weblogo.berkeley.edu/); 3D structures were predicted using Swiss Model online (http://swissmodel.expasy.org/).

## Results

### 1. Isolation and Analysis of the Novel *H^+^-PPase* Gene Sequences

**Figure 1:**
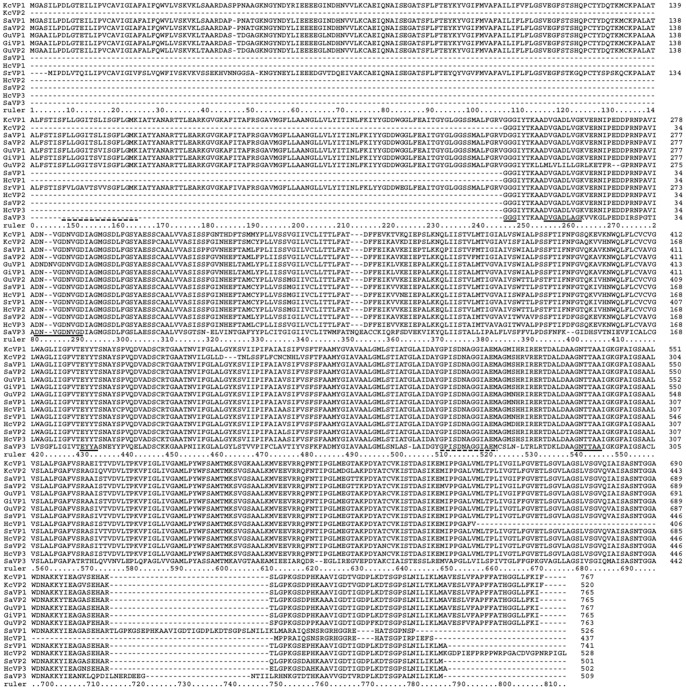
Multiple sequence alignment with H^+^-PPase sequences isolated from 7 eremophytes. Two ORFs and a fragment were from *Sa* (*SaVP1*, *SaVP2* and *SaVP3*). The two ORFs were from *Gu* (*GuVP1* and *GuVP2*) and one ORF from *Gi* (*GiVP1*). The two fragments were from *Ss* (*SsVP1* and *SsVP2*), one fragment was from *Sr* (*SrVP1*), and three fragments were from *Hc* (*HcVP1*, *HcVP2* and *HcVP3*). The other ORF and fragment were from *Kc* (*KcVP1* and *KcVP2*). The sequences marked with solid underline are the identified conserved domains GGG, DVGADLVGK, DNVGDNVGD, TEYYT, and GNTTAA [5], [25], [26]. The putative motifs GPISDNAGGIAEM and FLLGGITSLISGFLGM were marked by dotted underline.

**Figure 2:**
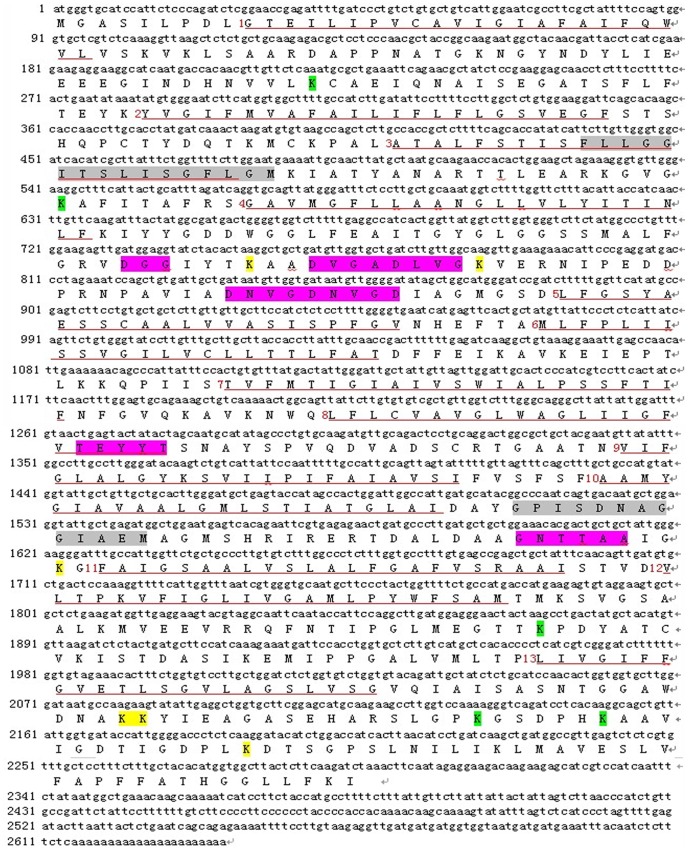
The sequence and transmembrane prediction of *SaVP1*. There are 13 transmembrane regions in *SaVP1* which are marked with red Arabic numerals. The yellow marked K is group I, and the green marked K is group II. The purple marked segment consists of identified conserved domains. The grey marked segment consists of supposed novel conserved motifs.

**Figure 3:**
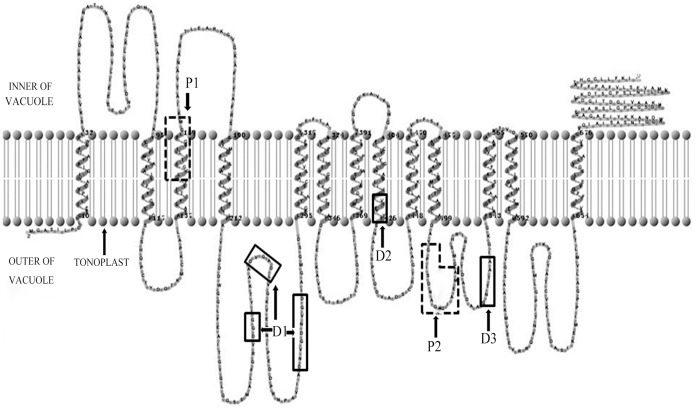
Tentative transmembrane model of H^+^-PPase from *Sophora alopecuroid* generated by TMHMM online. There are 13 putative transmembrane regions. D1, D2 and D3 with solid rectangles are previously identified domains [26], and P1 and P2 with dotted rectangles are putative motifs that we predicted.

**Figure 4:**
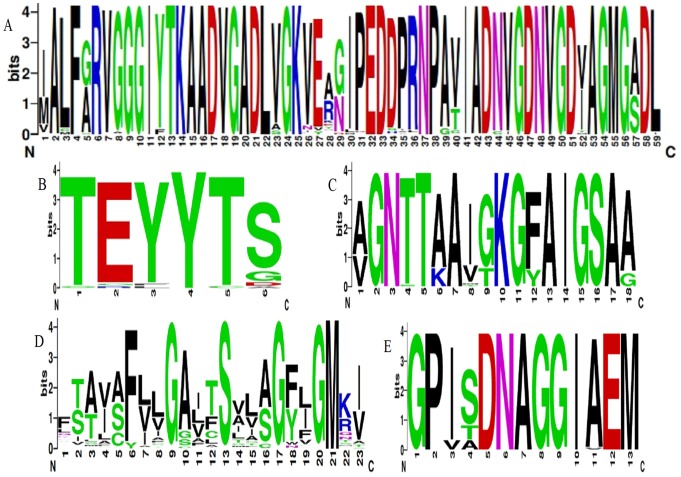
Consensus sequences of the conserved domains of H^+^-PPase that were isolated from 7 eremophytes and more than 240 H^+^-PPases from NCBI. The sequences IALFGRVDGGIYTKAADVGADLVGKVERNIPEDDPRNPAVIADNVG DNVGDIAGMGSDL (A), TEYYTS (B), and AGNTTAAIGKGFAIGSAA (C) are previously identified conserved domains [5], [26]. The sequences FTAVAFLLGGITS LISGFLGMKI (D) and GPISDNAGGIAEM (E) are new possible motifs, according to conserved sequence and structure.

A total of 6 complete ORF sequences and 8 conserved region fragments of H^+^-PPase were cloned from 7 donor eremophytes (Figure S1) by RT-PCR and the RACE method, respectively. Among them, 2 ORFs and 1 fragment were from *Sa* (named as *SaVP1*, *SaVP2* and *SaVP3*), 2 ORFs were from *Gu* (named as *GuVP1* and *GuVP2* ) and 1 ORF from *Gi* (named as *GiVP1*), while 2 fragments were from *Ss* (named as *SsVP1* and *SsVP2*), 1 fragment was from *Sr* (named as *SrVP1*), 3 fragments were from *Hc* (named as *HcVP1*, *HcVP2* and *HcVP3*) and the remaining ORF and fragment were from *Kc* (named as *KcVP1* and *KcVP2*). These amino acid sequences were aligned by CLUSTAL X (1.83) (Figure 1). In these six ORFs, the cDNA sequence of *GuVP1* and *KcVP1* contains 2304 bp and codes for 767 amino acids, the *SaVP1*, *SaVP2* and *GiVP1* genes contain 2298 bp and code for 765 amino acids, and the *GuVP2* gene contains 2292 bp and codes for 763 amino acids. The isoelectric point was 5.4±0.18 and the molecular weight approximately was 80.32±0.172 kD, as analyzed by the Compute pI/Mw tool (http://web.expasy.org/compute/), and 13 transmembrane regions were suggested by TMHMM (http://www.expasy.ch/tools/). The other 8 conserved region fragments are approximately 1500 bp and code for 500 amino acids except *SrVP1* which has 2223 bp and codes for 741 amino acids. The structures of these genes were analyzed through TMHMM software from Expasy online, using *SaVP1* as an example (Figure 2 and Figure 3). It was shown that the sequences from the 7 eremophytes are members of H^+^-PPase family as identified by BLASTx from NCBI online (http://blast.ncbi.nlm.nih.gov/Blast). These sequences have specific conserved domains of H^+^-PPase, namely the GGG, DVGADLVGK, and DNVGDNVGD domains [5], [26]. These specific conserved domains of H^+^-PPases are located between the 4th and 5th transmembrane region, which are exactly the same as reported except for *SaVP*1 with a DGG domain. The second conserved domain in all species is (E/D)YYT from 423–426 sites of *SaVP*1, located inside the 8th transmembrane region. The third domain is the K^+^-dependent conserved sequence GNTTAA [6], which is located near the 11th transmembrane region (Figure 4). These conserved domains are exposed to the cytoplasm (Figure 3). Based on the above characteristics, these genes from the 7 eremophytes belong to H^+^-PPase because their conserved domains are similar to the identified H^+^-PPases.

### 2. Homology Analysis of the H^+^-PPase between the 14 Novel H^+^-PPases and the Other H^+^-PPases from NCBI

Blast analysis showed that all of the novel *H^+^-PPase* genes, except for the *SaVP3* that were isolated from 7 eremophytes, have more than 70% sequence identity with the homologous *H^+^-PPase* genes that were identified from other plant species from NCBI (Figure S2). The highest sequence identity is 95% between *SaVP1*, *SaVP2*, *GuVP1*, *GuVP2* and *GiVP1* and vacuolar- type H^+^-pyrophosphatase (XM 003609415.1) from *Medicago truncatula*, and 95% between *KcVP*1, *KcVP*2 and *Glycine max* H^+^-PPase (XP_003542656.1). There was 96% identity between 6 novel genes from the three Chenopodiaceae plants and *Saliconia europaea* H^+^-PPase (AEI17666.1), while the identity was below 60% between *SaVP*3 and H^+^-PPase from higher plants and bacteria in the conserved region. The highest sequence identity was 64% between *SaVP3* and *Oxytricha trifallax* H^+^-PPase (EJY73348.1).

### 3. Comparison of Conserved Domains of H^+^-PPases

To compare the diversity of H^+^-PPases from different origins, we conducted an analysis on the homology of some specific conserved domains. Conserved domains of approximately 240 identified H^+^-PPases from NCBI and the 14 novel H^+^-PPase clones were analyzed. The conserved domains of *SaVP1* are as follows: 1. ALFGRVDGGIYTKAADVGADLVGKVERNIPEDDPRNPAVIADNVGDNVGDIAGMGSDL; 2. GFVTEYYTSNAYSP and 3. LDAAGNTTAAIGKGFA (the amino acid sequences underlined were conserved domains). Domain 1, with 57 amino acids, contains the GGG, DVGADLVGK and DNVGDNVGD domains of H^+^-PPase between the 4th and 5th transmembrane region (Figure 2) [5], [6], [26]. It has been reported that the GGG domain has three forms. Among them, GGG is the most popular one, and AGG/SGG is rare [26]; while DGG was only found in the *SaVP1*. DVGADLVGK and DNVGDNVGD, two nonapeptide sequences, are found among the 57 amino acids that are well conserved during evolution not only in prokaryote but also in eukaryote. Therefore, the three motifs in domain 1 were regarded as marked domains of H^+^-PPase (Figure 4A). The first nonapeptide sequence, DVGADLVGK, followed by the amino acids VE is similar not only in sequence but also in function to the plant vacuole sequence DX_7_KXE and bacterian sequence EX_7-8_KXE. Of all the novel H^+^-PPases from the 7 eremophytes, *GuVP2* does not have this motif (Figure 1). For the DNVGDNVGD domain of *GuVP1*, DN was repeated between DN and VG (Figure 1). There are rich G, A, D and V ‘very early’ amino acids [28] in the three motifs of domain 1, especially polar amino acid residues in DNVGDNVGD at positions 1, 5 and 9 of the nonapeptides. These three charge residues would be at the same surface with one turn every 3.6 amino acid residues.

Domain 2 contains EYYT and is located at the end of the 8th transmembrane region of *SaVP1* (Figure 2). The EYYT domain is conserved in all higher plants, while it is variable in every site in algae, protozoans, bacteria and archae (Figure 4B). The EYYT domain had a highly homology in all 14 novel eremophyte H^+^-PPases.

In all of the H^+^-PPases (more than 240 included) there is a greatly conserved GNTTAA domain (domain 3), except where 8 GNSTAA domains are found (Figure 4C). GNTTAA is considered to be an important H^+^-PPase domain because it could be related to potassium- dependence, especially due to the first A site [6], [29]. This site has two possible forms: A and K. A is a K^+^-dependent native and K is a K^+^-independent variant [6], [29]. In each of the 14 novel H^+^-PPases from 7 eremophytes, domain 3 exists as GNTTAA (Figure 1) and is located between the 10th and the 11th transmembrane region of *SaVP1* (Figure 2).

In addition to the 3 conserved domains mentioned above, Lee *et al*. [30] divided 18 lysines (K) of H^+^-PPase, which are in the cytosolic loop from *Vigna radiate*, into three groups according to the conserved K. Group I includes K^250^, K^261^, K^541^, K^694^, K^695^, and K^730^; these six K positions are highly conserved in the H^+^-PPases of higher plants and bacteria. Group II consists of moderately conserved lysines, such as K^73^, K^181^, K^624^, K^711^, and K^717^. Among these K sites, K^711^ and K^717^ are highly conserved in higher plants but not in prokaryotes. The lysines in group III are not conserved in the cytosolic loops. In all of the novel eremophyte H^+^-PPases, the position of K in group I and group II is similar (Figure 2) to those reported by Lee *et al*. (2011).

### 4. Hypothetical Convergent Evolutionary Position in the 7 Selected Eremophytes and the Other *H^+^-PPase* Genes that were Collected from NCBI

In addition to the reported conserved domains, novel conserved motifs were predicted in all the donated species. The first motif is FLLGGITSLISGFLGM which is located in the 3rd transmembrane end from amino acids 146 to 161 of *SaVP1* (Figure 2). The amino acids in positions 4, 8, 12, 15 and 16 of the motif especially are highly conserved in all species (Figure 4D). The underlined amino acids in the sequence GGITSLISG will be at the same surface based on the rule that α- helix has 3.6 residues per turn. Amino acids D, G and S are all polar amino acids, and the GGITSLISG structure is similar to the nonapeptide sequence of DNVGDNVGD in the 57 amino acid sequence mentioned above. Thus, this motif could have a vital function.

The other highly conserved motif contains 13 amino acid residues, which are located between the 10th and the 11th transmembrane region from amino acid 503 to 515 of the *SaVP1* (Figure 2). They formed GPISDNAGGIA EM, which was highly conserved in all of the species and is underlined (Figure 4E). Furthermore, in the motif GPISDNAGGIAEM, the “very early” amino acids G, D, and A account for 46%, while the polar amino acids account for 61%; this is similar to the 57 amino acid sequence in domain 1 mentioned above. They could be connected with vital structural, functional and evolutionary significance [26].

### 5. Phylogenetic Analysis between Novel Genes and the H^+^-PPases Identified from NCBI

**Figure 5:**
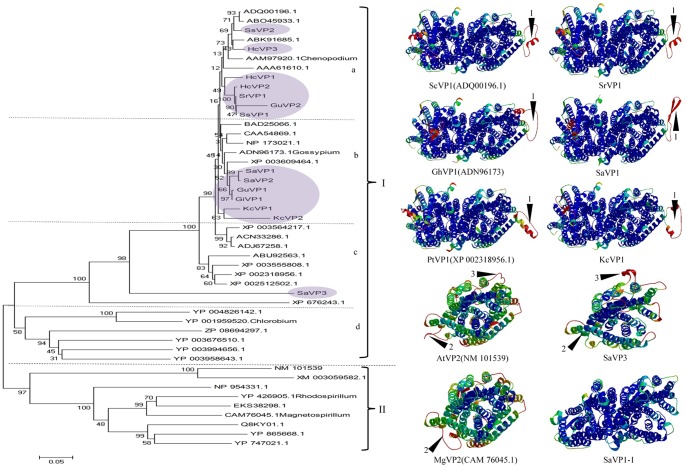
Phylogenetic tree of H^+^-PPase sequences from some representative species by NJ and a 3D structure prediction by Swiss Model. Purple indicates novel cloned H^+^-PPases from 7 eremophytes. The type I H^+^-PPases were made up of a, b, c and d subgroups. The a subgroup is made up of *ScVP* (ADQ00196.1), *HcVP* (ABO45933.1), *KfVP* (ABK91685.1), *ChrVP* (AAM97920.1), *SsVP2*, *HcVP3*, *MtVP* (XP_003609464.1), *SaVP1*, *SaVP2*, *GuVP1*, *GiVP1*, *GhVP* (ADN96173.1), *NtVP* (CAA54869.1), and *AVP1* (NP_173021.1). The b subgroup includes *OsVP* (BAD25066.1), *BvVP* (AAA61610.1), *HcVP*1, *HcVP*2, *SrVP*1, *SsVP*1, *GuVP*2, *KcVP1* and *KcVP2*. The c subgroup is formed by *BdVP* (XP_003564217.1), *ZmVP* (ACN33286.1), *SbVP* (ADJ67258.1), *ZxVP* (ABU92563.1), *GmVP* (XP_003555808.1), *PtVP* (XP_002318956.1), *RcVP* (XP_002512502.1). And the d is formed by *ChlrVP* (XP_001694682.1), SaVP3, *PbVP* (XP_676243.1), *RhmVP* (YP_004826142.1), *ChpVP* (YP_001959520.1), *ThmVP* (YP_003676510.1), *ElVP* (YP_003958643.1), *HhVP* (YP_003994656.1), *FvVP* (ZP_08694297.1). The type II H^+^-PPase includes *AVP2* (NM_101539), *MpVP* (XM_003059582.1), *RhrVP* (YP_426905.1), *MgVP* (CAM76045.1), *NeVP* (YP_747021.1), *GsVP* (NP_954331.1), *AbVP* (EKS38298.1), *RhpVP* (Q8KY01.1), and *MmVP* (YP_865668.1). 3D structures including type I H^+^-PPase *ScVP1* (ADQ00196.1, *Suaeda corniculata*), *OsVP1* (BAD25066.1, *Oryza sativa*), *ChrVP1* (XP_001694682.1, *Chlamydomonas reinhardtii*), *SrVP1*, *SaVP1* and *KcVP1* are present as homodimers. The others are monomer H^+^-PPase, they are *AVP2* (NM_101539, *Arabidopsis thaliana 2*), *SaVP3* and *MgVP2* (CAM76045.1, *Magnetospirillum gryphiswaldense*). The *SaVP1-1* was truncated according to the sequence of *SaVP3*. Number 1, 2 and 3 and black arrows in the 3D structure showed different regions.

To explore the phylogenetic relationship between the novel *H^+^-PPase* genes from the selected 7 eremophytes and the H^+^-PPase identified from NCBI, we constructed a phylogenetic tree using the CLUSTAL X1.83 and NJ methods. This was performed for 34 *H^+^-PPase* genes from 33 species, which included 25 type I and 9 type II *H^+^-PPase* genes. Among the 25 type I *H^+^-PPase* genes, 17 *H^+^-PPase* genes were from higher plants, including Chenopodiaceae, Leguminosae and Gramineae, and other 8 type I *H^+^-PPase* genes were from algae, protozoans and bacteria. The genetic relationship of these 33 species and 7 eremophytes was displayed in Figure S3 by referring to some reports [31]. The phylogenetic analysis indicated these *H^+^-PPase* genes were divided into 2 major clusters: type I and type II H^+^-PPase. The novel clones of 14 *H^+^-PPase* genes from 7 eremophytes were marked with purple and were clustered into type I H^+^-PPase in the phylogenetic tree (Figure 5). *H^+^-PPase* genes from different species were clustered into different subgroups. It was shown that 2 novel *H^+^-PPase* genes, *SsVP2* and *HcVP3* were clustered into the a-subgroup with the H^+^-PPases identified from Chenopodiaceae, which included *ScVP*, *HcVP*, *KfVP* and *ChrVP.* Four novel *H^+^-PPase* genes from Leguminosae, including *SaVP1*, *SaVP2*, *GuVP1* and *GiVP1*, were clustered together with *MtVP*. These H^+^-PPases were clustered into the a-subgroup with *AVP1*, *NtVP*, and *GhVP*. The other 4 novel *H^+^-PPase* genes from Chenopodiaceae, *HcVP1*, *HcVP2*, *SrVP1* and *SsVP1*, as well as *GuVP2* from *Glycyrrhiza uralensis*, were clustered into the b-subgroup with *BvVP* from *Beta vulgris*, *OsVP* from *Oryza sativa*, and *KcVP1* and *KcVP2* from Compositae *Karelinia caspia*. Four genes from monocotyledoneae, including *BdVP*, *ZmVP*, *SbVP*, and *ZxVP*, were classified together with *GmVP*, *PtVP*, and *RcVP* from dicotyledoneae into the c-subgroup. *SaVP3* from *Sophora alopecuroides* was located between the H^+^-PPases of *Chlamydomonas reinhardtii* and *Plasmodium berghei*; these together with other H^+^-PPases from bacteria, including *ChlrVP*, *PbVP*, *RhmVP*, *ChpVP*, *ThmVP*, *ElVP*, *HhVP* and *FvVP*, formed the d-subgroup. All of the above, a, b, c and d subgroups formed the set of type I H^+^-PPases. The set of type II H^+^-PPases was made up of *AVP2*, *MpVP*, *RhrVP*, *MgVP*, *NeVP*, *GsVP*, *AbVP*, *RhpVP*, and *MmVP*.

### 6. Prediction of H^+^-PPase 3D Structure

In addition to the molecular evolution of H^+^-PPases, we were interested in the structural similarity among H^+^-PPase proteins. To understand the structural similarities and variations within the H^+^-PPase family, we used homology modeling to build 3D structures of H^+^-PPase members using the Swiss Model. It was shown that all H^+^-PPases were rich in α-helixes. The type I H^+^-PPase which were from higher plants that were homodimers included *ScVP1*, *SrVP1*, *OsVP1*, *SaVP1*, *ChrVP1* and *KcVP1*, while *SaVP3*, *AVP2*, and *MgVP1* were monomers (Figure 5). To check the accuracy of the *SaVP3* 3D structure, *SaVP1* was truncated to make it comparable to *SaVP3* because *SaVP3* was not a complete ORF. It was shown that the truncated *SaVP1* was a homodimer as *SaVP1-1* (Figure 5), which implied that the length of amino acids did not change the 3D structure of *SaVP1*. Though we did not obtain the full sequence of *SaVP3*, the accordance between *SaVP1* and the truncated *SaVP1* indirectly meant that the predicted 3D structure of *SaVP3* monomer should be correct. To confirm the structure of H^+^-PPase of bacteria, the 3D structures of 10 bacterial H^+^-PPases were predicted. The predicted structure showed that native H^+^-PPases consisted of monomers in bacteria (Figure S4). Meanwhile, the predicted 3D structures of H^+^-PPases showed that all type I H^+^-PPases in higher plants consisted of homodimers [32], while both type I H^+^-PPases in bacteria and protozoans and all type II H^+^-PPases consisted of monomers.

Although there are high similarities among type I H^+^-PPases at the primary amino acid structure, there is a difference at the 3D structures. The difference was found at position 1, as noted by black arrows in Figure 5. The structure noted with black arrow was shown as α-helix and random coil at the position 1 of type I H^+^-PPases from Chenopodiaceae, *Oryza sativa*, *Chlamydomonas reinhardtii* and *Karelinia caspia*, but it was a β-fold in the *Sophora alopecuroides* H^+^-PPase. Monomeric H^+^-PPases are clearly different at position 2 (Figure 5), such as a β-corner in the type II H^+^-PPase of *A. thaliana*, a random coil in *Magnetospirillum gryphiswaldense*, or a β-fold in *Sophora alopecuroides SaVP3*. In summary, for the same protein, different species may have different protein structures.

## Discussion

H^+^-PPases are important enzymes during PPi-hydrolyzing and PPi-energizing H^+^ translocation, with highly conserved sequences. The H^+^-PPase is not only present in the vacuolar membrane of plants but also in the acidocalcisome membrane; it provides H^+^, rich PPi and polyP, and indirectly accumulates calcium and other elements [9], [15]. However, because the acidocalcisome was regarded as an ancestral organelle which possesses ancestral physiological function in both prokaryotes and eukaryotes, H^+^-PPase was considered to be an ancestral gene [7]. Thus, there is little diversity at the amino acid sequence of H^+^-PPase, implying that these genes play a vital role in the development of the organism and have suffered little selection pressure. In this report, we cloned 14 H^+^-PPases from 7 eremophytes, in which 13 H^+^-PPases shared 70–96% identity with other H^+^-PPases that were identified from higher plants, all of which are available on NCBI except *SaVP3*, the E values approach zero.

In the amino acid sequence of H^+^-PPase ALFGRVGGGIYTKAADVGADLVGKVERNIPEDDPRNPAVIADNVGDNVGDIAGMGSDL, EYYT and GNTTAA motifs were regarded as specific domains of the H^+^-PPase family [25], [26]. These motifs could be related to Mg-PPi binding, PPi hydrolysis and energy transfer [33]. It was hypothesized that the GGG sequence provides a spacer to allow mimicking of the swing of the lever arm of a myosin motor [5], [34], and this sequence may change the mechanism for the physiological coupling between the light-induced pumping of protons and either the photophosphorylation of inorganic phosphate (Pi) to PPi or the hydrolysis of PPi to Pi under dark condition [4]. This motif consisted of DGG in *SaVP1* and it has other forms, such as AGG and SGG, it implying that GGG was substitutable at the first site. DVGADLVGKVE and DNVGDNVGD were considered to participate directly in substrate (Mg- PPi) binding and/or hydrolysis [26], [35] or Mg^2+^ or Ca^2+^ binding based on the 3D structure of soluble PPases and related to the origin of the gene [24]. *GuVP2* does not have the DVGADLVGKVE domain (Figure 1), while DN was repeated in the DNVGDNVGD domain of *GuVP1* as DNDNVGDNVGD. These could be affected by environment. The other H^+^-PPases were highly conserved in the GGG, DVGADLVGKVE and DNVGDNVGD domains (Figure 4A). These domains could be related to the evolution origin because they are rich in G, A, D and V, which are ‘very early’ amino acids in these three domains [6], [26], [28]. These characteristics of the H^+^-PPase domains appear to be of vital structural, functional and evolutionary significance [24].

EYYT is a highly conserved domain in higher plants. Similarly, this domain was highly conserved in the 14 novel clones of the H^+^-PPases from eremophytes. EYYT was inferred to play a role in coupling PPi hydrolysis and could be related to H^+^ translocation and H^+^-PPase activity in plant vacuoles [25], [36]. The GNTTAA domain is considered to be a marker of type I/II, according to the first A site [6]. This site has two possible forms, A and K. It was believed that the A form is the K^+^- dependent H^+^-PPase and the K form is K^+^-independent H^+^-PPase [6], [29], [37]. This domain is GNTTAA in all 14 novel eremophyte H^+^-PPases. In the thermophilic bacterium *Carboxydothermus hydrogenoformans* the A^460^/K^460^ position is occupied by Ala in the K^+^-dependent H^+^-PPase and by Lys in the K^+^-independent H^+^-PPase, while the G^463^ (Ala)/T^463^ position is occupied by G or A in the K^+^-dependent H^+^-PPases and T in the K^+^-independent H^+^-PPases [6], [37]. It was found that an A460K substitution in *C. hydrogenoformans* H^+^-PPase is sufficient to confer K^+^ independence to both PPi hydrolysis and PPi-energized H^+^ translocation. In contrast, the A463T mutation does not affect the K^+^-dependence of H^+^-PPase [37]. This suggested that the classification of H^+^-PPase could have other evidence. It was believed that type I H^+^-PPase was located in vacuolar membranes and that the type II H^+^-PPase was located in Golgi membranes [25], [35], [38].

Phylogenic analysis showed that sequence identity was related to a genetic relationship: in the a-subgroup, the H^+^-PPases from Chenopodiaceae were clustered together and others from Leguminosae were clustered together (Figure 5). This phenomenon of type I H^+^-PPases from diverse species clustering together indicated that they could be orthologous genes [39]. At the same time, there was convergent evolution in the b-subgroup, in which 6 novel clones of H^+^-PPases from eremophytes, including Chenopodiaceae, Leguminosae and Compositae, were clustered together (Figure 5). This could be the result of environmental selection over the long term. Besides, there were a few genes, such as *SaVP3* and *AVP2*, the identity is 66% between *SaVP3* and *SaVP1* in the coverage, and it is only 36% between *AVP2* and *AVP1* [20]. Their sequence identity had little relation to their genetic relationship, type I and type II H^+^-PPases in the same species could be paralogous genes [39].

Using radiation inactivation and gel permeation HPLC, Sato *et al*. [32] and Chanson and Pilet [40] showed that the H^+^-PPases of pumpkin and maize were dimmers, though previous reports showed the H^+^-PPases in Mung Bean [41] and red beet [42] were a single polypeptide by SDS-PAGE analysis. The reason might be the protein was not native in SDS-PAGE [32]. These conclusions suggested that type I H^+^-PPases were structurally similar and existed as dimers in higher plants. In our study, we predicted the 3D structure of H^+^-PPase from different species and the result showed that type I H^+^-PPases from higher plants and algae were homodimers, which was conformed to experimental results in higher plants. Other type I H^+^-PPases from bacteria and protists and all type II H^+^-PPases existed as monomers.

Specially, *SaVP3* is type I H^+^-PPase based on the conserved domain GNTTAA. However, compared with *SaVP1* and other H^+^-PPases, the amino acid sequence identify is very low. Moreover, phylogenetic analysis showed that *SaVP3* was clustered together with *ChrVP* of *Chlamydomonas reinhardtii* and *PbVP* of *Plasmodium berghei*. 3D structure analysis showed that both *SaVP3* and *AVP2* were present as monomers, and their amino acid sequence identity were low with *SaVP1* and *AVP1* in the same species, respectively. It is possible that different evolutionary histories or lateral gene transfers of H^+^-PPase between different (micro) organisms exist [20], [25]. Because of the attribution impossibility of *SaVP3* into type I/II H^+^-PPase, it was inferred that *SaVP3* might be the evolutionary origin of *SaVP1* in *Sa*, or directly was a novel type H^+^-PPase. Our result imply that the 3D structure prediction could be a novel approach for homology protein classification.

### Conclusion

In this report, 14 *H^+^-PPase* genes were cloned from 7 eremophytes and their sequences were compared with more than 240 other identified H^+^-PPase available in the NCBI. At the same time, a phylogenic tree was constructed with 14 novel cloned genes and 34 representations of H^+^-PPase sequences. We inferred that H^+^-PPases could have 2 other conserved motifs in addition to the 3 identified conserved domains. These highly conserved motifs indicates that H^+^-PPase plays an important role during the development of the organism and is also characterized by a small amount of influence from the environment during evolution. The 3D structures of some of the H^+^-PPases were also predicted. It was shown that type I H^+^-PPases from higher plants are homodimers, while type I H^+^-PPases from bacteria and protozoans and all type II H^+^-PPases are present as monomers. This regularity motif and structure could provide important evidence on evolutionary origin of H^+^-PPase and in the study of the relationship between its structure and function.

## Supporting Information

Figure S1 **The selected 7 eremophytes and their natural habitats.** These plants were in Alar environs. A: *Sophora alopecuroid* L. (*Sa*), B: environment of these plants in winter, C: *Glycyrrhiza uralensis Fisch* L. (*Gu*), D: *Glycyrrhiza inflata Batalin* L. (*Gi*), E: *Suaeda salsa* L. (*Ss*), F: S*uaeda rigida* Kung et G. L. (*Sr*), G: *Halostachys caspica* L. (*Hc*), and H: *Karelinia caspia* (Pall.) L. (*Kc*).
(TIF)

Figure S2 **Multiple sequence alignment of the H^+^-PPase amino acid sequences from 14 novel cloned H^+^-PPases and 34 previously identified H^+^-PPase that were selected from NCBI.** Their name and accession number of these identified H^+^-PPases are the same as in Figure 5.
(TIF)

Figure S3 **The genetic relationship of the selected species.** The species phylogenic tree was constructed for the 34 identified sequences of H^+^-PPase from 33 representative species, in which 2 sequences were from *Arabidopsis thaliana*. The 7 eremophytes marked with red are the selected donor species.
(TIF)

Figure S4 **Predicted 3D structure of the H^+^-PPases in bacteria and protozoans.** The gene name and accession number of these identified H^+^-PPases are the same as in Figure 5.
(TIF)

## References

[1] Bassil E, Tajima H, Liang YC, Ohto M, Ushijima K, et al (2011) The Arabidopsis Na^+^/H^+^ antiporters NHX1 and NHX2 control vacuolar pH and K^+^ homeostasis to regulate growth, flower development, and reproduction. Plant Cell 23: 3482–3497. 21954467 10.1105/tpc.111.089581 PMC3203450

[2] Hasegawa PM, Bressan RA, Zhu JK, Bohnert HJ (2000) Plant Cellular and Molecular Responses to High Salinity. Annu Rev Plant Physiol Plant Mol Biol 51: 463–499. 15012199 10.1146/annurev.arplant.51.1.463

[3] Yang H, Knapp J, Koirala P, Rajagopal D, Peer WA, et al (2007) Enhanced phosphorus nutrition in monocots and dicots over-expressing a phosphorus- responsive type I H^+^-pyrophosphatase. Plant Biotechnol J 5: 735–745. 17711412 10.1111/j.1467-7652.2007.00281.x

[4] Baltscheffsky H, Von Stedingk LV, Heldt HW, Klingenberg M (1966) Inorganic pyrophosphate: formation in bacterial photophosphorylation. Science 153: 1120–1122. 4288237 10.1126/science.153.3740.1120

[5] Baltscheffsky M, Schultz A, Baltscheffsky H (1999) H^+^-proton-pumping inorganic pyrophosphatase: a tightly membrane-bound family. FEBS Lett 452: 121–127. 10386575 10.1016/s0014-5793(99)00617-1

[6] Serrano A, Perez-Castineira JR, Baltscheffsky M, Baltscheffsky H (2007) H^+^-PPases: yesterday, today and tomorrow. IUBMB Life 59: 76–83. 17454298 10.1080/15216540701258132

[7] Docampo R, Ulrich P, Moreno SNJ (2010) Evolution of acidocalcisomes and their role in polyphosphate storage and osmoregulation in eukaryotic microbes. Philosophical Transactions of the Royal Society B: Biological Sci 365: 775–784. 10.1098/rstb.2009.0179 PMC2817225 20124344

[8] Lipmann F, Fox S (1965) The origins of pre-biological systems and of their molecular matrices. New York: Academic Press, 361–382 p.

[9] Seufferheld MJ, Kim KM, Whitfield J, Valerio A, Caetano-Anolles G (2011) Evolution of vacuolar proton pyrophosphatase domains and volutin granules: clues into the early evolutionary origin of the acidocalcisome. Biol Direct 6: 50–64. 21974828 10.1186/1745-6150-6-50 PMC3198990

[10] López-Marqués RL, Pérez-Castiñeira JR, Losada M, Serrano A (2004) Differential regulation of soluble and membrane-bound inorganic pyrophosphatases in the photosynthetic bacterium Rhodospirillum rubrum provides insights into pyrophosphate-based stress bioenergetics. J bacteriology 186: 5418–5426. 10.1128/JB.186.16.5418-5426.2004 PMC490873 15292143

[11] Baltscheffsky NGHH (2011) Links Between Hydrothermal Environments, Pyrophosphate, Na^+^, and Early Evolution. Orig Life Evol Biosph: 41 (5): 485–495. 10.1007/s11084-011-9235-4 PMC3178022 21461648

[12] Baltscheffsky M (1967) Inorganic pyrophosphate and ATP as energy donors in chromatophores from Rhodospirillum rubrum. Nature 216: 241–243. 4293681 10.1038/216241a0

[13] Baltscheffsky M (1969) Reversed energy conversion reactions of bacterial photophosphorylation. Arch Biochem Biophys 133: 46–53. 4309363 10.1016/0003-9861(69)90486-x

[14] Docampo R, Moreno SN (2011) Acidocalcisomes. Cell Calcium 50: 113–119. 21752464 10.1016/j.ceca.2011.05.012 PMC3156361

[15] Seufferheld M, Vieira MC, Ruiz FA, Rodrigues CO, Moreno SN, et al (2003) Identification of organelles in bacteria similar to acidocalcisomes of unicellular eukaryotes. J Biol Chem 278: 29971–29978. 12783865 10.1074/jbc.M304548200

[16] Perez-Castineira JR, Alvar J, Ruiz-Perez LM, Serrano A (2002) Evidence for a wide occurrence of proton-translocating pyrophosphatase genes in parasitic and free-living protozoa. Biochem Biophys Res Commun 294: 567–573. 12056804 10.1016/S0006-291X(02)00517-X

[17] Motta LS, Ramos IB, Gomes FM, de Souza W, Champagne DE, et al (2009) Proton-pyrophosphatase and polyphosphate in acidocalcisome-like vesicles from oocytes and eggs of Periplaneta americana. Insect Biochem Molec Biol 39: 198–206. 19111615 10.1016/j.ibmb.2008.11.003

[18] Perez-Castineira JR, Lopez-Marques RL, Losada M, Serrano A (2001) A thermostable K^+^-stimulated vacuolar-type pyrophosphatase from the hyperther- mophilic bacterium Thermotoga maritima. FEBS Lett 496: 6–11. 11343697 10.1016/s0014-5793(01)02390-0

[19] Baltscheffsky M, Nadanaciva S, Schultz A (1998) A pyrophosphate synthase gene: molecular cloning and sequencing of the cDNA encoding the inorganic pyrophosphate synthase from Rhodospirillum rubrum. Biochim Biophys Acta 1364: 301–306. 9630689 10.1016/s0005-2728(98)00062-0

[20] Drozdowicz YM, Kissinger JC, Rea PA (2000) AVP2, a sequence-divergent, K^+^-insensitive H^+^-translocating inorganic pyrophosphatase from Arabidopsis. Plant Physiol 123: 353–362. 10806252 10.1104/pp.123.1.353 PMC59009

[21] McIntosh MT, Drozdowicz YM, Laroiya K, Rea PA, Vaidya AB (2001) Two classes of plant-like vacuolar-type H^+^-pyrophosphatases in malaria parasites. Mol Biochem Parasitol 114: 183–195. 11378198 10.1016/s0166-6851(01)00251-1

[22] Sarafian V, Kim Y, Poole RJ, Rea PA (1992) Molecular cloning and sequence of cDNA encoding the pyrophosphate-energized vacuolar membrane proton pump of Arabidopsis thaliana. Proc Natl Acad Sci U S A 89: 1775–1779. 1311852 10.1073/pnas.89.5.1775 PMC48535

[23] Meng X, Xu Z, Song R (2011) Molecular cloning and characterization of a vacuolar H^+^-pyrophosphatase from Dunaliella viridis. Mol Biol Rep 38: 3375–3382. 21086174 10.1007/s11033-010-0445-z

[24] Mitsuda N, Enami K, Nakata M, Takeyasu K, Sato MH (2001) Novel type Arabidopsis thaliana H^+^-PPase is localized to the Golgi apparatus. FEBS Lett 488: 29–33. 11163790 10.1016/s0014-5793(00)02400-5

[25] Drozdowicz YM, Rea PA (2001) Vacuolar H^+^-pyrophosphatases: from the evolutionary backwaters into the mainstream. Trends in plant sci 6: 206–211. 11335173 10.1016/s1360-1385(01)01923-9

[26] Hedlund J, Cantoni R, Baltscheffsky M, Baltscheffsky H, Persson B (2006) Analysis of ancient sequence motifs in the H^+^-PPase family. FEBS J 273: 5183–5193. 17054711 10.1111/j.1742-4658.2006.05514.x

[27] Zhu LF, Tu LL, Zeng FC, Liu DQ, Zhang XL (2005) An improved simple protocol for isolation of high quality RNA from Gossypium spp. suitable for cDNA library construction. Acta Agronomica Sinica 31: 1657–1659.

[28] Baltscheffsky H, Persson B, Schultz A, Pérez-Castineira J, Baltscheffsky M (2004) Origin and evolution of very early sequence motifs in enzymes. Life in the universe Kluwer Academic Publishers, Dordrecht, The Netherlands: 107–110.

[29] Hirono M, Mimura H, Nakanishi Y, Maeshima M (2005) Expression of functional Streptomyces coelicolor H^+^-pyrophosphatase and characterization of its molecular properties. J Biochem 138: 183–191. 16091593 10.1093/jb/mvi112

[30] Lee CH, Pan YJ, Huang YT, Liu TH, Hsu SH, et al (2011) Identification of essential lysines involved in substrate binding of vacuolar H^+^-pyrophosphatase. J Biol Chem 286: 11970–11976. 21292767 10.1074/jbc.M110.190215 PMC3069399

[31] Jiao Y, Wickett NJ, Ayyampalayam S, Chanderbali AS, Landherr L, et al (2011) Ancestral polyploidy in seed plants and angiosperms. Nature 473: 97–100. 21478875 10.1038/nature09916

[32] Sato MH, Maeshima M, Ohsumi Y, Yoshida M (1991) Dimeric structure of H^+^-transllocating pyrophosphatase from pumpkin vacuolar membranes. FEBS J 290: 177–180. 10.1016/0014-5793(91)81254-6 1655530

[33] Gaxiola RA, Palmgren MG, Schumacher K (2007) Plant proton pumps. FEBS Lett 581: 2204–2214. 17412324 10.1016/j.febslet.2007.03.050

[34] Suzuki Y, Yasunaga T, Ohkura R, Wakabayashi T, Sutoh K (1998) Swing of the lever arm of a myosin motor at the isomerization and phosphate-release steps. Nature 396: 380–383. 9845076 10.1038/24640

[35] Rea PA, Britten CJ, Sarafian V (1992) Common identity of substrate binding subunit of vacuolar H^+^-translocating inorganic pyrophosphatase of higher plant cells. Plant Physiol 100: 723–732. 16653052 10.1104/pp.100.2.723 PMC1075619

[36] Zhen RG, Kim EJ, Rea PA (1997) Acidic residues necessary for pyrophosphate-energized pumping and inhibition of the vacuolar H^+^-pyrophosphatase by N, N'- dicyclohexylcarbodiimide. J Biol Chem 272: 22340–22348. 9268385 10.1074/jbc.272.35.22340

[37] Belogurov GA, Lahti R (2002) A lysine substitute for K^+^. A460K mutation eliminates K^+^ dependence in H^+^-pyrophosphatase of Carboxydothermus hydrogenoformans. J Biol Chem 277: 49651–49654. 12401795 10.1074/jbc.M210341200

[38] Rea PA, Kim Y, Sarafian V, Poole RJ, Davies JM, et al (1992) Vacuolar H^+^-translocating pyrophosphatases: a new category of ion translocase. Trends Biochem Sci 17: 348–353. 1329278 10.1016/0968-0004(92)90313-x

[39] Koonin EV (2005) Orthologs, paralogs, and evolutionary genomics 1. Annu Rev Genet 39: 309–338. 16285863 10.1146/annurev.genet.39.073003.114725

[40] Chanson A, Pilet PE (1989) Target molecular size and sodium dodecyl sulfate-polyacrylamide gel electrophoresis analysis of the ATP- and pyrophosphate-dependent proton pumps from maize root tonoplast. Plant Physiol 90: 934–938. 16666900 10.1104/pp.90.3.934 PMC1061823

[41] Maeshima M, Yoshida S (1989) Purification and properties of vacuolar membrane proton-translocating inorganic pyrophosphatase from mung bean. J Biol Chem 264: 20068–20073. 2555340

[42] Britten CJ, Turner JC, Rea PA (1989) Identification and purification of substrate-binding subunit of higher plant H^+^-translocating inorganic pyrophosphatase. FEBS Lett 256: 200–206.

