# Supplementary figures and images for: Isolation and Characterization of a Conserved Domain in the Eremophyte H+-PPase Family
Source: PLoS One. 2013 Jul 29;8(7):e70099. doi: 10.1371/journal.pone.0070099 (PMC3726567; doi:10.1371/journal.pone.0070099)

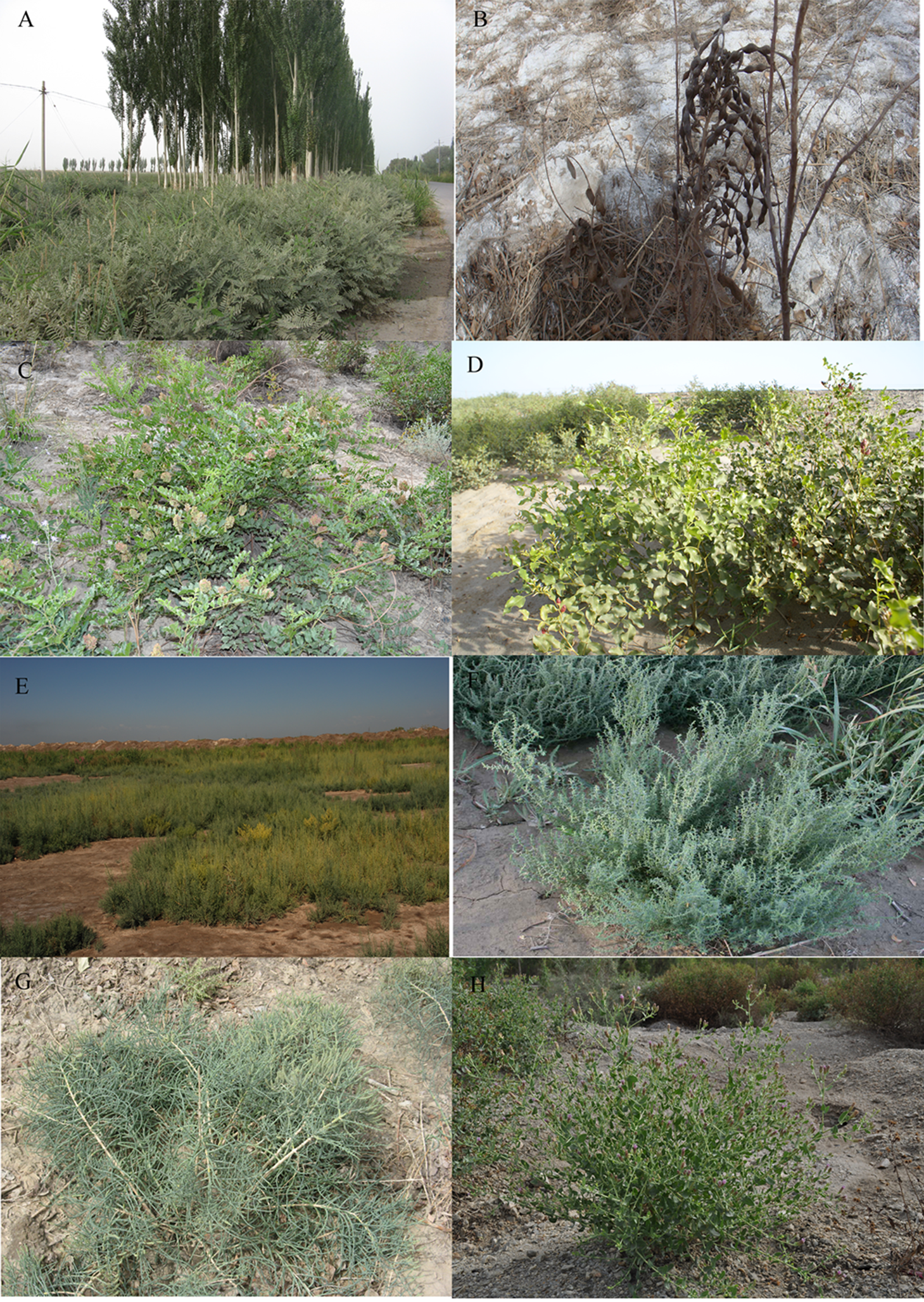

Supplement: Figure S1 — The selected 7 eremophytes and their natural habitats. These plants were in Alar environs. A: Sophora alopecuroid L. (Sa), B: environment of these plants in winter, C: Glycyrrhiza uralensis Fisch L. (Gu), D: Glycyrrhiza inflata Batalin L. (Gi), E: Suaeda salsa L. (Ss), F: Suaeda rigida Kung et G. L. (Sr), G: Halostachys caspica L. (Hc), and H: Karelinia caspia (Pall.) L. (Kc). (TIF) [file pone.0070099.s001.tif]

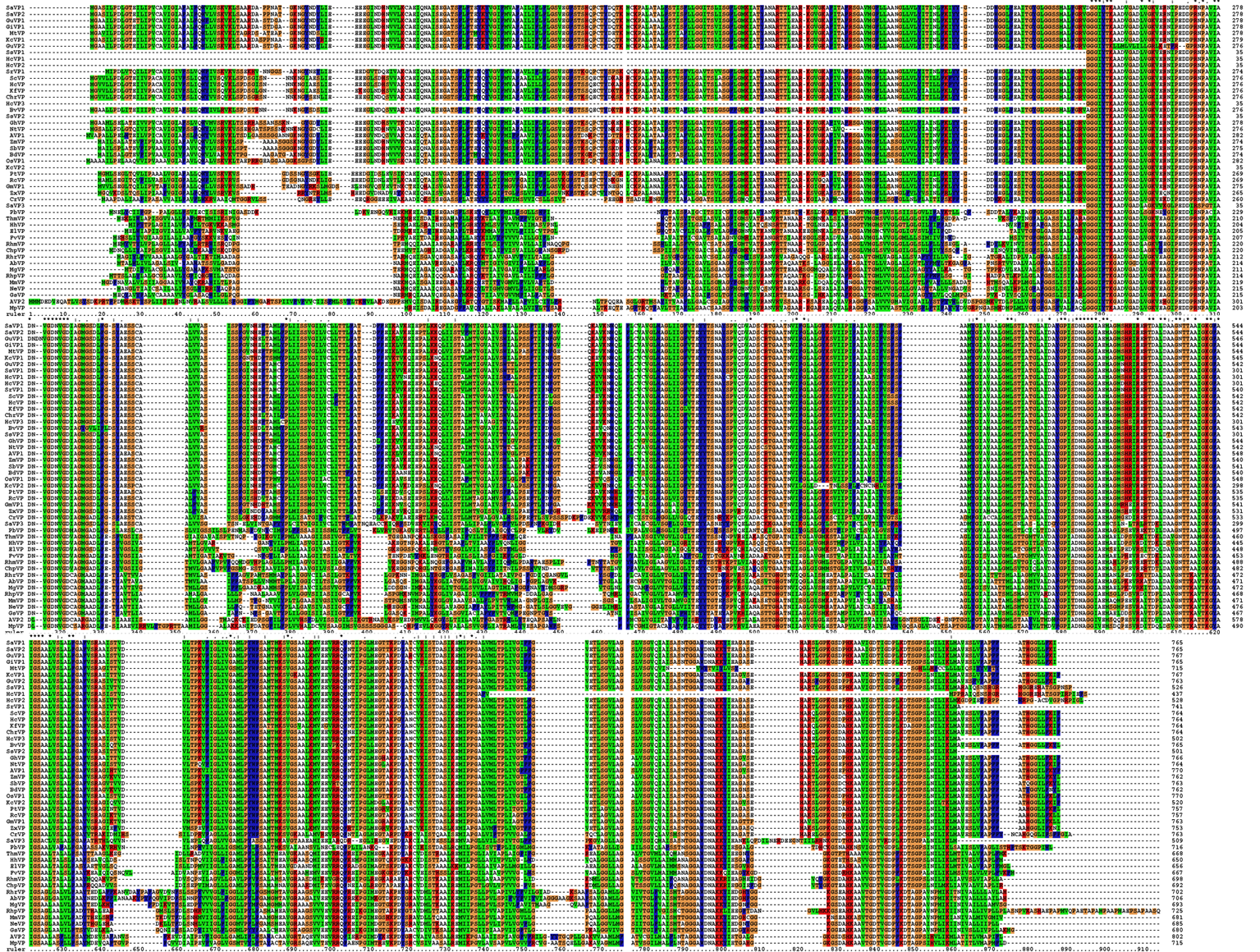

Supplement: Figure S2 — Multiple sequence alignment of the H+-PPase amino acid sequences from 14 novel cloned H+-PPases and 34 previously identified H+-PPase that were selected from NCBI. Their name and accession number of these identified H+-PPases are the same as in Figure 5. (TIF) [file pone.0070099.s002.tif]

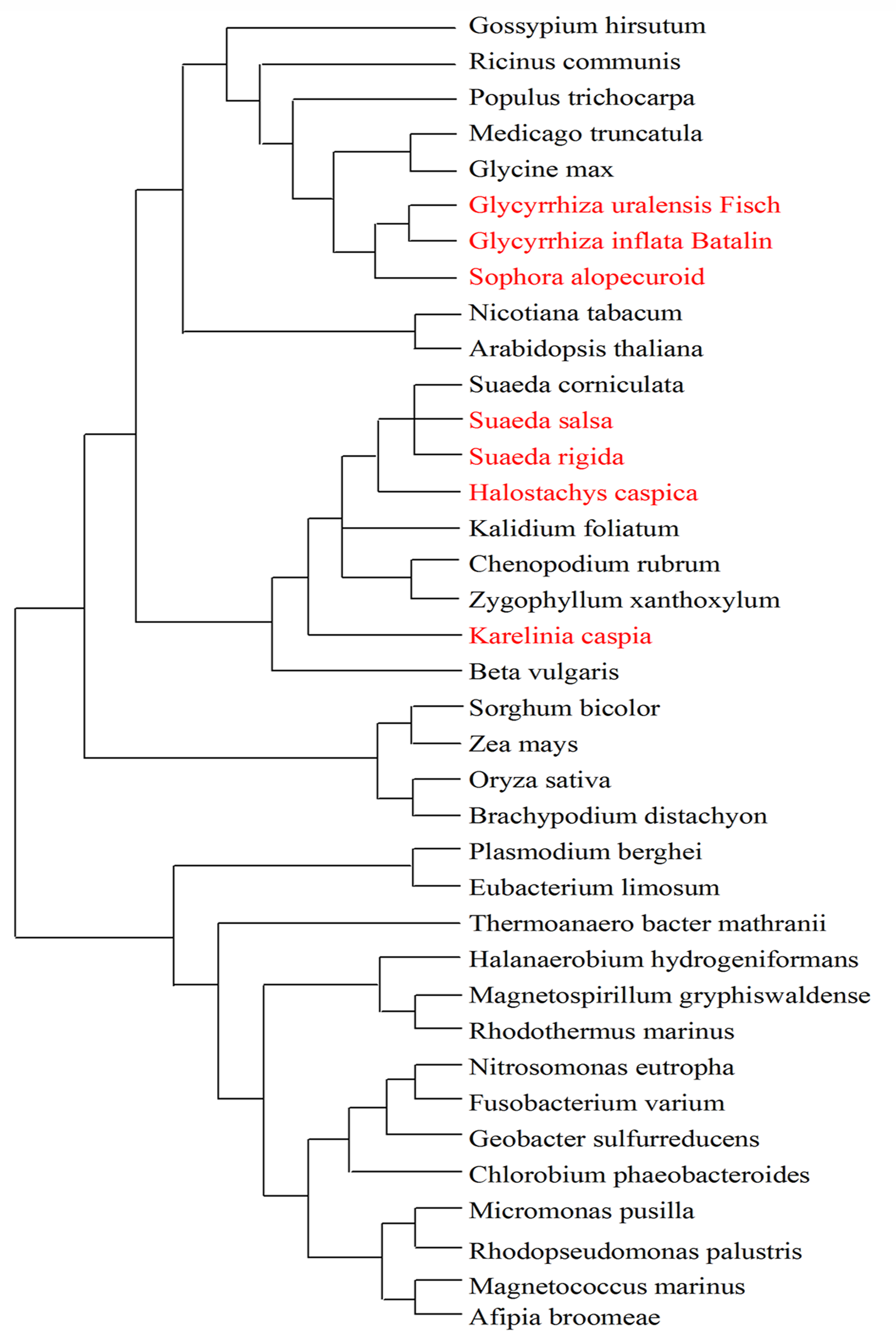

Supplement: Figure S3 — The genetic relationship of the selected species. The species phylogenic tree was constructed for the 34 identified sequences of H+-PPase from 33 representative species, in which 2 sequences were from Arabidopsis thaliana. The 7 eremophytes marked with red are the selected donor species. (TIF) [file pone.0070099.s003.tif]

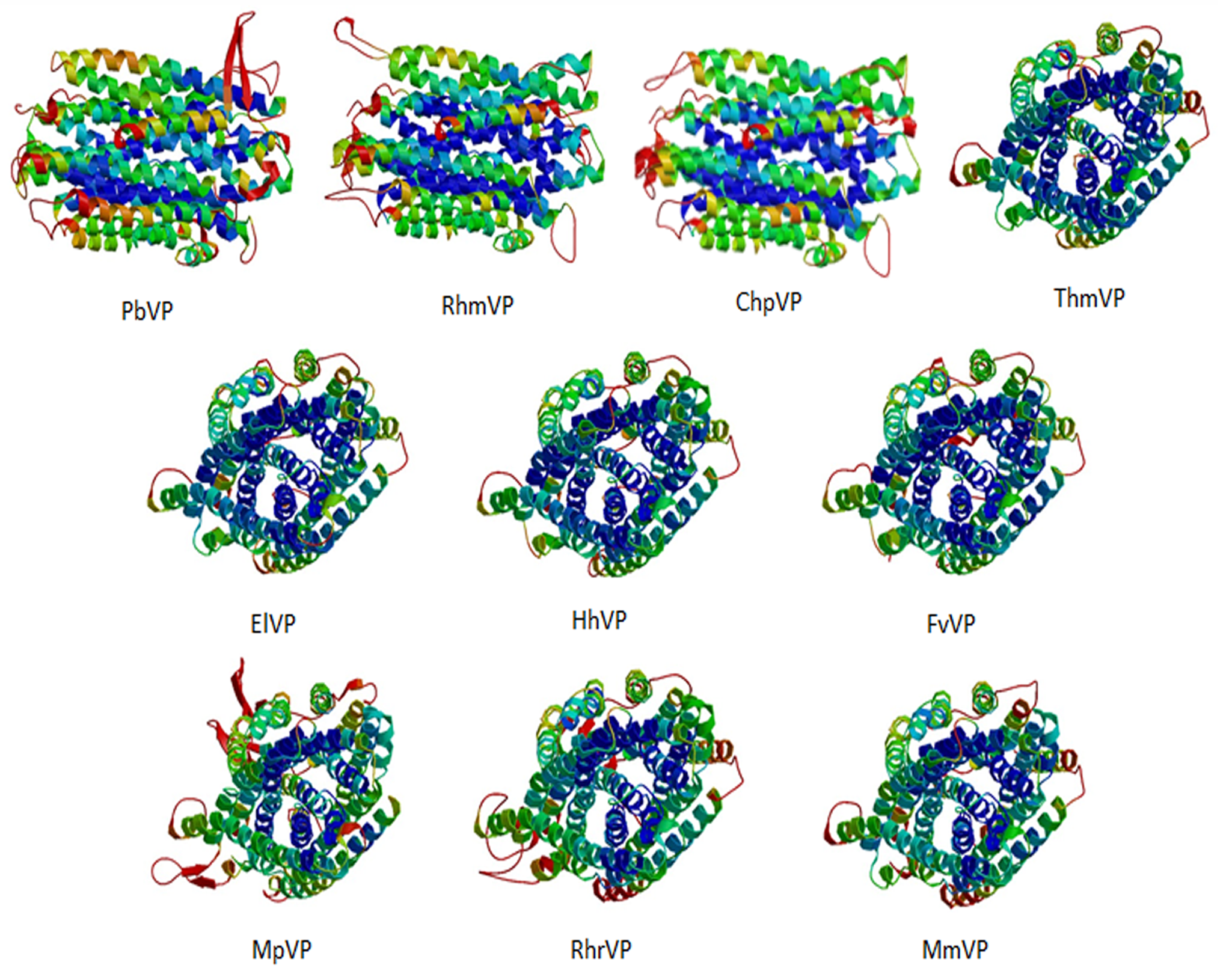

Supplement: Figure S4 — Predicted 3D structure of the H+-PPases in bacteria and protozoans. The gene name and accession number of these identified H+-PPases are the same as in Figure 5. (TIF) [file pone.0070099.s004.tif]
